# Macrophage-2-Binding Protein Glycosylation Isomer (M2BPGi) and AGAP Score as Markers of Noninvasive Test for Liver Fibrosis versus FibroScan in Chronic Hepatitis B Patients: A Retrospective Observational Study

**DOI:** 10.1155/2024/6635625

**Published:** 2024-05-29

**Authors:** Laila Kurnia Pramono, Anna Tjandrawati, Dewi Kartika Turbawaty, Tiene Rostini, Muhammad Begawan Bestari, Haryono, Deny Budiman, Prapanca Nugraha

**Affiliations:** ^1^ Department of Clinical Pathology Faculty of Medicine Universitas Padjadjaran, Bandung, Indonesia; ^2^ Department of Clinical Pathology Faculty of Medicine Dr. Hasan Sadikin General Hospital, Bandung, Indonesia; ^3^ Department of Internal Medicine Faculty of Medicine Dr. Hasan Sadikin General Hospital, Bandung, Indonesia; ^4^ Department of Surgery Faculty of Medicine Universitas Padjadjaran, Bandung, Indonesia

## Abstract

**Background:**

Liver biopsy as the gold standard for assessing the degree and diagnosis of fibrosis still has significant drawbacks, which make the emergence of a much less invasive diagnostic marker possible. M2BPGi levels and the AGAP score, the two newest serological markers, are known to have good sensitivity for detecting liver fibrosis. This study is aimed at determining the validity of examining M2BPGi levels and AGAP scores on the Fibroscan examination as markers of noninvasive test for liver fibrosis in chronic hepatitis B patients.

**Methods:**

This is an observational, descriptive study with a retrospective design. This study used secondary data taken from medical records and blood specimen research materials of outpatients at the Hepatology Gastroenterology Polyclinic at a tertiary general hospital in West Java, Indonesia, with a diagnosis of chronic hepatitis B.

**Results:**

There were 109 research subjects included. There were 73 (66.9%) subjects with no- or low-grade fibrosis and 36 (33.1%) with advanced fibrosis. The sensitivity and specificity of the M2BPGi were 88.9% and 61.6% (PPV 55.3%; NPV 91.8%; AUC 0.753), while the AGAP score was 47.2% and 100% (PPV 100%; NPV 79.3%; AUC 0.736). The combined M2BPGi level and the AGAP score showed a sensitivity of 80.9% and a specificity of 100% (PPV 100%; NPV 91.8%; AUC 0.905).

**Conclusion:**

The AGAP score and M2BPGi levels together are a better way to measure the degree of liver fibrosis in people with chronic hepatitis B than either M2BPGi or the AGAP score alone.

## 1. Introduction

Hepatitis B is a disease caused by the hepatitis B virus that occurs in the liver. Based on WHO estimates, in 2019, it is estimated that as many as 296 million people will have chronic hepatitis B, with 1.5 million new infections each year [1]. In the United States, there are 60,000 new cases of HBV infection each year, with 2 million more people having chronic hepatitis B infection. In Indonesia, based on the 2019 Ministry of Health, the number of people with hepatitis B reached 20.3% and was ranked third in the number of HBV infections after China and India [2]. The body's immune response due to a hepatitis B virus infection results in an inflammatory process that causes liver damage. This process plays a role in the progression of liver disease into cirrhosis or hepatocellular carcinoma [3]. Liver fibrosis is the accumulation of interstitial or extracellular matrix (MES) scar tissue after acute or chronic liver injury. The categorization of fibrotic conditions ranges from noncirrhotic (stages F0-F3) to cirrhotic (stage F4) [4–7]. Examination methods used to diagnose liver fibrosis can be invasive, noninvasive, or a combination of both [8]. A liver biopsy examination is the gold standard for assessing the degree and diagnosis of fibrosis. The drawbacks of liver biopsy are related to the high cost, invasive procedures, availability of competent multidisciplinary experts and personnel, and various complications. Routinely repeating liver biopsies to monitor the progress of fibrosis is also not efficient in daily practice, so a combination of noninvasive tests is needed [4]. Noninvasive tests include physical approaches such as measurement of liver stiffness using Fibroscan® and biological approaches such as examination of serum biomarkers. Being the newest serological marker, M2BPGi is one of the glycoprotein markers that is being widely studied and is thought to function for early detection in the early stages of fibrosis. The AGAP score is also included in the latest noninvasive tests published in early 2022. The AGAP score uses the parameters AST, gamma-glutamyl transferase (GGT), age, and platelet count in its calculations [9]. This study is aimed at determining the validity of examining M2BPGi levels and AGAP scores on the Fibroscan examination as markers of noninvasive liver fibrosis in chronic hepatitis B patients.

## 2. Methods

### 2.1. Study Design and Setting

**Figure 1:**
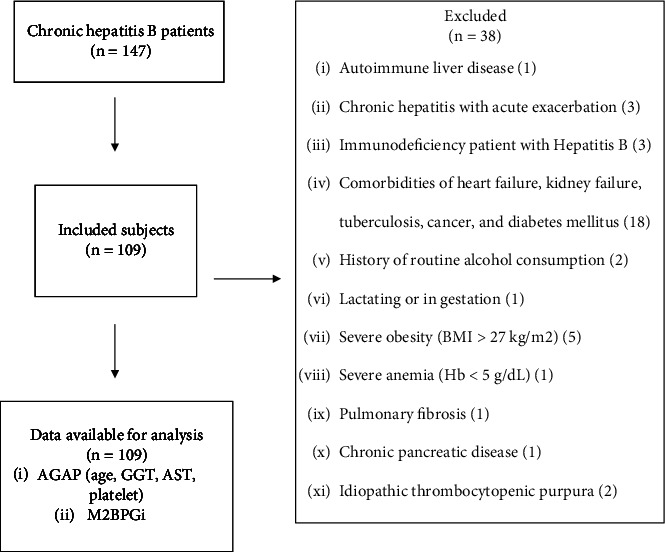
The flowchart of the study selection process.

This research is a retrospective, cross-sectional study and follows the Strengthening the Reporting of Observational Studies in Epidemiology (STROBE) guidelines [10]. The method used is a diagnostic test to assess the sensitivity, specificity, positive predictive value (PPV), and negative predictive value (NPV) of examining M2BPGi levels and AGAP scores. This study used secondary data taken from medical records of outpatients at the Hepatology Gastroenterology Polyclinic at a tertiary general hospital in West Java, Indonesia, with a diagnosis of chronic hepatitis B from January to December 2023. The sample size was formulated for a descriptive quantitative outcome from Lemeshow and Lwanga and Egbuchulem with a minimum size of 97 [11, 12]. The inclusion criteria include being 18 years old and being HBsAg seropositive for more than six months (declared to have chronic hepatitis B). Exclusion criteria are shown in Figure 1, and at least one is on the list. This research was done in accordance with the Declaration of Helsinki, and the hospital's ethical committee approved this study with a registered number of LB.02.01/X.6.5/262.

### 2.2. Data Collection

Patient characteristics, fibrosis level, M2BPGi, and AGAP score details were extracted from the medical record. The blood sample was taken from the patient on the same day as the Fibroscan examination. Patient characteristics that were collected were sex, age, status of antiviral therapy, hepatitis B virus e antigen (HBeAg), platelet count, AST, and GGT level.

### 2.3. Statistical Analysis

The collected data were analyzed using the Statistical Package for Social Science (SPSS) for Windows version 26.0 program. Each quantitative variable is written as a percentage, and its numerical value is listed as the mean, standard deviation, or median. Validity tests were presented as sensitivity, specificity, positive predictive value, negative predictive value, and area under the receiver operating characteristic (ROC) curve.

### 2.4. Definition of Variables

#### 2.4.1. M2BPGi Examination Procedure

The examination of M2BPGi levels was carried out using a two-step sandwich immunoassay method using chemiluminescence. The automatic test equipment measures M2BPGi using 10 *μ*l of serum sample with a cut-off point index (COI); M2BPGi <1 means negative and >1 means positive, as recommended by Baudi et al. [13] and Inoue and Tanaka [14]. A two-step sandwich immunoassay uses chemiluminescence. Principles of Inspection Serum M2BPGi levels are detected using a specific lectin called *Wisteria floribunda* agglutinin (WFA), which recognises N-acetygalactosamine residues of N-glycans and O-glycans on M2BP. The specific lectin will be immobilised using magnetic beads, mixed with diluted serum. Monoclonal antibodies in the form of anti-M2BP labelled with ALP are then added after washing the unbound proteins. After that, chemiluminescent substrate and stop solution are added to remove unbound antibodies before fluorescent reading. The automated assay measures M2BPGi using 10 *μ*l of a serum sample within 17 minutes. The cut-off point index (COI) of M2BPGi is reported using the HISCL-5000 or 800 immunoanalyzer made by Sysmex Corp., Hyogo, Japan, with a range of 0.1–20, and a value < 1.0 is considered negative in light intensity units.

The whole blood sample in the SST tube must be left until a complete blood clot occurs. The sample was then centrifuged at 3000 rpm for 10 minutes to separate serum and blood cells.

Samples in SST tubes can be used directly for examination. The tool system will automatically carry out the following things: dilute the sample with reagent R1. WFA coated with magnetic particles (MP) in reagent R2 will react specifically with the glycosylated isomer of M2BP present in the sample. Washing (B/F separation) is carried out, and then the ALP-labelled anti-M2BP monoclonal antibodies (mouse) in the R3 reagent will bind specifically with M2BPGi on MP. Washing is carried out, and then the ALP in the MP is decomposed with the substrate.

CDP-Star, contained in reagent R5, then produces a luminescence signal.

#### 2.4.2. The AGAP Score Calculation

The AGAP score is calculated using the AGAP score formulation: [AST level (U/L) × GGT level (U/L)] × [age (years)/platelet count (10^9^/L)^2^], where the AGAP score is ≤4.038, which means negative, and >4.038, which means positive, as recommended by Okdemir and Cakmak [9]. Platelet count examination used whole blood specimens in tubes with EDTA anticoagulant and was carried out using the XN-1000 hemology analyzer. Platelet count examination uses the optical flow cytometry method using the RET channel. The lysis reagent slightly perforates the cell membranes of erythrocytes, leukocytes, and platelets so that the fluorescence penetrates the cells. Fluorescence labels intracellular nucleic acids, where the intensity of the resulting fluorescence signal is directly proportional to the nucleic acid content in platelets. Examination of AST levels uses the Wrobleswki and LaDue methods, which are modified with serum examination materials. This method uses pyridoxal-5-phosphate (P5P) as an enzymatic reaction activator. The stability of the inspection material is 3 days at a temperature of 20–250°C, 7 days at a temperature of 2–80°C, and 1 month at a temperature of -800°C. The examination of the GGT level method is an adaptation of the methodology recommended by the IFCC (International Federation of Clinical Chemistry and Laboratory). This method uses the substrate L-gamma-glutamyl with glycylglycine. The examination material is serum, stable for 8 hours at 20–250°C, 2 days at 2-80°C, and -200°C or colder for longer storage.

#### 2.4.3. Fibroscan Examination

The fibrosis level was measured using the Fibroscan for measuring liver stiffness, and the Fibroscan measurement result was expressed in kPa. The Fibroscan examination procedure is carried out on patients lying down supine with the right arm slightly raised, and then the probe is placed on the surface of the skin around the 9th to 11th ribs. This procedure uses the Doppler technique; the speed of the shear wave (s-wave) is delivered through the liver parenchyma. When operating the probe, the operator will press a button to start measurements (shots). Fibroscan examination results can be said to be valid if they have achieved 10 successful shots with a median interquartile ratio of less than 0.3. Median tissue stiffness values are grouped based on the degree of liver fibrosis according to the METAVIR score, with the classifications F0-F4: F0-F1 (<6 kPa); F2 (6.1–8.9 kPa); F3 (9–11.9 kPa); and F4 (>12 kPa). No- or low-grade fibrosis was F0 through F2, and advanced fibrosis was F3 and F4.

## 3. Discussion

Data on the characteristics of the study subjects showed that males did not differ much from females with liver fibrosis due to chronic hepatitis B in the group of no- or low-grade fibrosis subjects. This could be due to the unequal number of research subjects between men and women, in contrast to the advanced fibrosis subject group, which shows slightly more males than females. This is in accordance with the study of Mak et al., which found that the number of subjects with liver fibrosis due to chronic hepatitis B was higher in males (70%) [15]. This could be because men are more often exposed to risk factors for transmission of the hepatitis B virus infection, for example, lifestyle influences (such as sexually transmitted infections, injecting drug use, and tattoos) compared to women [16, 17]. The mean age of the study subjects with no- or low-grade fibrosis was 39 ± 12 years, and with advanced fibrosis, it was 52 ± 13 years. This is in accordance with the study of Nakamura et al. in 2017, who obtained the age range of chronic hepatitis B subjects, namely 25–68 years [18].

Based on Table 1, most of the study subjects had received antiviral therapy, and as many as 77% (84 subjects) had negative HBeAg. The results of this study showed that there were more HBeAg-negative subjects in both groups with no- or low-grade fibrosis and advanced fibrosis compared to HBeAg-positive subjects. The results of this study are in accordance with the research of Widita et al. in 2010, which stated that out of 105 serum samples of chronic hepatitis B patients as research subjects, there were more HBeAg negative samples, as many as 80 samples, namely 76.19% (consisting of negative HBeAg and anti-HBe positive, which was more dominant (82.5%) compared to negative HBeAg and anti-HBe negative) [19]. Research subjects who have not received antiviral therapy and have negative HBeAg can be caused naturally by mutations in the hepatitis B virus that do not produce HBeAg (negative HBeAg with positive anti-HBe). In this study, there was no complete examination data for study subjects regarding anti-HBe or HBV DNA, so the possibility of viral mutations or the phases of chronic hepatitis B infection could not be investigated further. The mean number of platelets in the no/low-grade fibrosis subject group in this study is higher than the advanced. This is in accordance with the study of Zhong et al., who found that the median platelet count decreased with worsening liver fibrosis (*F*0 = 221, *F*1 = 210, *F*2 = 188, *F*3 = 171, and F4 = 155.5 thousand/*u*L) [20].

In this study, the median AST levels in both groups were within normal limits, but the group of subjects with advanced fibrosis had a median AST and GGT level higher than the group of subjects with no/low-grade fibrosis. This is consistent with the study of Okdemir and Cakmak, who found that the median AST and GGT levels were higher in advanced fibrosis, although they did not exceed twice the upper limit of the normal value (median AST no/low-grade fibrosis 34 (24-61.5) U/L and advanced fibrosis 53 (36-93) U/L) and the median GGT level no/low-grade fibrosis 27.5 (16-50) U/L and advanced fibrosis 73 (39-117) U/L) [9]. Based on the characteristics of the study subjects (Table 1), positive M2BPGi results with advanced fibrosis were higher than those with no/low-grade fibrosis. Research by Mak et al. showed that M2BPGi levels correlated with the degree of liver fibrosis and showed excellent accuracy, especially in diagnosing severe fibrosis (AUC *F*3 = 0.795 and *F*4 = 0.914) [15]. However, in mild fibrosis (F0/F1), serum M2BPGi levels are relatively low and may appear normal. The AGAP score in Table 1 shows a negative result in more no/low-grade fibrosis (100%) than advanced fibrosis (52.8%). This is in accordance with the results of a study by Okdemir and Cakmak, which showed that subjects with a low degree of fibrosis or who did not experience liver fibrosis (F0-F2) had a negative AGAP score [9].

**Figure 2:**
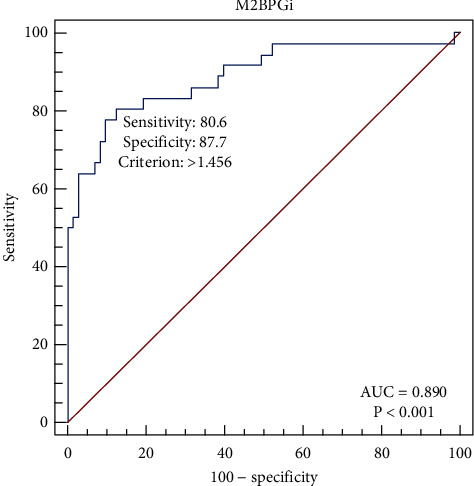
Range of curve analysis for M2BPGi levels and levels on the Fibroscan® examination.

The results of the validity test for testing M2BPGi levels in Table 2 show that testing for M2BPGi levels has a high sensitivity (88.9%) and low specificity (61.6%) for determining the presence of liver fibrosis. The data from our study show that range of curve (ROC) analysis for M2BPGi as shown in Figure 2 gave a high sensitivity of 80.6% and a high specificity of 87.7% for the cut-off value of >1.456, with an AUC of 0.890 (*p* < 0.001). Our data suggest the use of a higher cut-off value (>1.456) compared to a cut-off of 1, as recommended by Baudi et al. [13] and Inoue and Tanaka [14], giving lower sensitivity but higher specificity. This is in accordance with the study by Tsuji et al. in 2020 in Japan, which found that examination of M2BPGi levels in subjects with liver fibrosis due to chronic hepatitis B has a sensitivity ranging from 75.7 to 99.1% [21]. However, examination of M2BPGi levels has low specificity to be able to differentiate subjects without liver fibrosis. This is in accordance with a study by Hur et al. in Korea, which showed that examination of M2BPGi levels had a low specificity (50%) in liver fibrosis due to chronic hepatitis B with a degree of fibrosis ≤ *F*2, but a high specificity (92.6%) in the degree of fibrosis F3-F4 [22].

The positive predictive value (PPV) of examining M2BPGi levels in Table 2 shows that only 53.3% of subjects who underwent testing for M2BPGi levels with positive results actually had liver fibrosis. Based on Table 3, the results of the validity test for examining M2BPGi levels in subjects who had not received antiviral therapy had a sensitivity of 84.6%, a specificity of 61.3%, a PPV of 47.8%, an NPV of 90.5%, and an AUC of 0.730. In subjects who had received antiviral therapy, the results of the validity test for examining M2BPGi levels increased slightly, with a sensitivity of 91.3%, a specificity of 61.9%, a PPV of 56.8%, an NPV of 92.8%, and an AUC of 0.766. Several studies have shown that antiviral therapy is effective (at least for at least 1 year) to reduce M2BPGi levels in patients with liver fibrosis due to chronic hepatitis B and has been shown to decrease M2BPGi levels as liver function improves [23].

In this study, examination of M2BPGi levels showed good validity, especially in assessing severe fibrosis (F3–F4). Research by Nah et al. states that although not as high on the degree of fibrosis F3/F4, the significance of serum M2BPGi on the degree of fibrosis F0-F2 is still quite good [24]. Based on Table 4, all subjects in the no/low-grade fibrosis group showed a negative AGAP score, so there were no false positive results. A total of 19 subjects in the advanced fibrosis group had a negative AGAP score, resulting in false negative results. A study by Long et al. suggested that the use of antiviral therapy or a combination of antiviral therapy with hepatoprotective drugs in patients with liver fibrosis due to chronic hepatitis B can improve liver function by reducing elevated levels of liver enzymes (AST, ALT, GGT, and bilirubin) [25]. The research by Surana et al. showed that HBV DNA levels initially had a median of 7.0 (5.6–8.1) log IU/mL and decreased to 5.7 (4.3–6.5) log IU/mL after 1 year of antiviral therapy, while platelets do not affect the effect of antiviral therapy with a median baseline platelet count of 182 (156–205) thousand/uL. After 1 year of antiviral therapy, it becomes 170 (156–191) thousand/uL [26].

**Figure 3:**
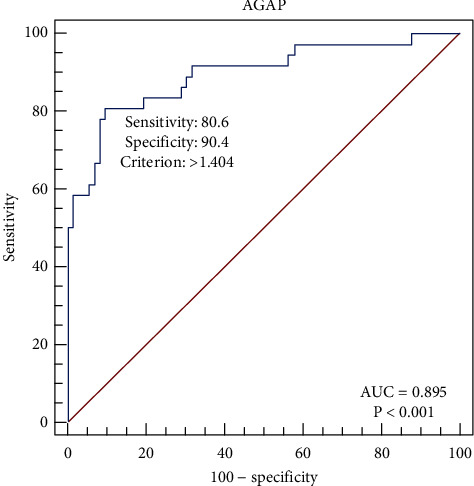
Range of curve analysis for AGAP score on the Fibroscan® examination.

Based on Table 4, the AGAP score has a sensitivity of 47.2%, a specificity of 100%, a PPV of 100%, an NPV of 79.3%, and an AUC of 0.736. The interesting thing is that the AGAP score has very high specificity and PPV (100%), so it is expected that the AGAP score can be used as a confirmatory marker to determine the degree of liver fibrosis in chronic hepatitis B patients. The NPV AGAP score of 79.3% is probably due to the high false-negative rate on this examination. The data from our study show that range of curve (ROC) analysis for AGAP score as shown in Figure 3 gave a high sensitivity of 80.6% and a high specificity of 90.4% for the cut-off value of >1.404, with an AUC of 0.895 (*p* < 0.001). Our data suggest the use of a lower cut-off value (>1.404) compared to a cut-off of >4.038, as recommended by Okdemir and Cakmak [9], giving higher sensitivity but lower specificity. In Table 5, the AGAP score still has a low sensitivity but very high specificity (100%). The NPV value of the AGAP score in subjects who had not received antiviral therapy was higher (83.8%) than the NPV score in subjects who had received antiviral therapy (76.4%). The liver enzymes used in calculating the AGAP score (AST and GGT) tend to be normal or not high in subjects who have received antiviral therapy. When we use patients who have shown positive M2BPGi levels and a positive AGAP score, the combined result shows higher sensitivity and higher specificity, as shown in Tables 6 and 7.  Table 7 shows that having a history of antiviral therapy in research subjects can increase the sensitivity of the combined examination to 83.3% with an AUC of 0.917.

The limitation of this study was that the number of research subjects was not balanced between no/low-grade fibrosis and advanced fibrosis, which affected the validity of the M2BPGi examination results and the AGAP score. In addition, research subjects with negative HBeAg status due to mutations of the hepatitis B virus could not be traced further, and this study was only conducted in one hospital (a single center). The combined examination of M2BPGi levels and AGAP score can be used as an alternative to laboratory-based noninvasive tests to determine the degree of liver fibrosis in chronic hepatitis B patients. Data from this study also show that a higher cut-off value for M2BPGi levels (>1.456) and a lower cut-off value for the AGAP score (>1.404) will give higher sensitivity and higher specificity. Further studies are needed regarding the validity of the M2BPGi, AGAP scores, and their cut-off values to monitor the effectiveness of antiviral therapy in improving liver function because studies showed improvement in the liver as seen from these two parameters after being given antiviral therapy.

## 4. Conclusion

Examination of M2BPGi levels has high sensitivity but low specificity in estimating the extent of hepatic fibrosis in those with chronic hepatitis B. The AGAP score has high specificity but low sensitivity in estimating the extent of hepatic fibrosis in those with chronic hepatitis B. The combined examination of M2BPGi levels and the AGAP score has good validity in estimating the extent of hepatic fibrosis in those with chronic hepatitis B patients compared to the single parameter M2BPGi or AGAP score. Having a history of antiviral therapy does not increase the validity of the AGAP score. As clinicians, we can utilise the measurement of the M2BPGi or AGAP score for patients with hepatitis B as a screening procedure for the evaluation of the fibrosis level as an alternative to a noninvasive laboratory-based examination.

## Figures and Tables

**Table 1:** Characteristics of research subjects.

Characteristics	No/low-grade fibrosis *n* = 73	Advanced fibrosis *n* = 36
Sex, *n* (%)		
Male	38 (52.1)	21 (58.3)
Female	35 (47.9)	15 (41.7)
Age (years), mean ± SD	39 ± 12	52 ± 13
Status of antiviral therapy, *n* (%)		
Received therapy	42 (57.5)	23 (63.9)
Have not received therapy	31 (42.5)	13 (36.1)
HBeAg, *n* (%)		
Positive	19 (26.0)	6 (16.7)
Negative	54 (74.0)	30 (83.3)
Platelet count (thousands/uL), mean ± SD	262 ± 72	183 ± 78
AST (U/L), median (IQR)	23 (20–28)	37 (27–56)
GGT (U/L), median (IQR)	28 (21–34)	58 (35–92)
M2BPGi		
Negative	45 (61.6)	4 (11.1)
Positive	28 (38.4)	32 (88.9)
AGAP score		
Negative	73 (100.0)	19 (52.8)
Positive	0 (0.0)	17 (47.2)

Note: AST: aspartate aminotransferase; GGT: gamma-glutamyl transferase; M2BPGi: Mac-2-binding protein glycosylation isomer; AGAP score: AST, GGT, age, and platelet.

**Table 2:** Validity test of the examination of M2BPGi levels on the Fibroscan® examination.

`	Grade Fibroscan (METAVIR Fibroscan)		
	Advanced fibrosis	No/low-grade fibrosis	Sensitivity: 88.9% Specificity: 61.6% Positive predictive value (PPV): 53.3% Negative predictive value (NPV): 91.8% Area under the ROC curve (AUC): 0.753
	*n* = 36	*n* = 73	
M2BPGi			
Positive (COI ≥ 1)	32	28	
Negative (COI < 1)	4	45	

Note: M2BPGi: glycosylated isomer of Mac-2-binding protein).

**Table 3:** Validity test for examination of M2BPGi levels on the Fibroscan® examination in subjects who have not received or have received antiviral therapy.

	**`**	Grade Fibroscan (METAVIR Fibroscan)		
		Advanced fibrosis	No/low-grade fibrosis	
Have not received antiviral therapy	M2BPGi	*n* = 13	*n* = 31	Sensitivity: 84.6% Specificity: 61.3% Positive predictive value (PPV): 47.8% Negative predictive value (NPV): 90.5% Area under the ROC curve (AUC): 0.730
	Positive (COI ≥ 1)	11	12	
	Negative (COI < 1)	2	19	
Have received antiviral therapy	M2BPGi	*n* = 23	*n* = 42	Sensitivity: 91.3% Specificity: 61.9% Positive predictive value (PPV): 56.8% Negative predictive value (NPV): 92.8% Area under the ROC curve (AUC): 0.766
	Positive (COI ≥ 1)	21	16	
	Negative (COI < 1)	2	26	

**Table 4:** Validity test of the AGAP score on the Fibroscan® examination.

**`**	Grade Fibroscan (METAVIR Fibroscan)		
	Advanced fibrosis	No/low-grade fibrosis	Sensitivity: 47.2% Specificity: 100% Positive predictive value (PPV): 100% Negative predictive value (NPV): 79.3% Area under the ROC curve (AUC): 0.736
	*n* = 36	*n* = 73	
AGAP score			
Positive (>4.038)	17	0	
Negative (≤4.038)	19	73	

Note: AGAP score: AST, GGT, age, and platelet.

**Table 5:** Validity test for the AGAP score on the Fibroscan® examination in subjects who have not received or have received antiviral therapy.

	**`**	Grade Fibroscan (METAVIR Fibroscan)		
		Advanced fibrosis	No/low-grade fibrosis	
Have not received antiviral therapy	AGAP score	*n* = 13	*n* = 31	Sensitivity: 53.8% Specificity: 100% Positive predictive value (PPV): 100% Negative predictive value (NPV): 83.8% Area under the ROC curve (AUC): 0.769
	Positive (>4.038)	7	0	
	Negative (≤4.038)	6	31	
Have received antiviral therapy	AGAP score	*n* = 23	*n* = 42	Sensitivity: 43.5% Specificity: 100% Positive predictive value (PPV): 100% Negative predictive value (NPV): 76.4% Area under the ROC curve (AUC): 0.717
	Positive (>4.038)	10	0	
	Negative (≤4.038)	13	42	

**Table 6:** Combined validity test examination of M2BPGi levels and the AGAP score on the Fibroscan® examination.

`	Grade Fibroscan (METAVIR Fibroscan)		
	Advanced fibrosis	No/low-grade fibrosis	Sensitivity: 80.9% Specificity: 100% Positive predictive value (PPV): 100% Negative predictive value (NPV): 91.8% Area under the ROC curve (AUC): 0.905
	*n* = 21	*n* = 45	
M2BPGi + AGAP score			
Positive	17	0	
Negative	4	45	

Note: positive results: M2BPGi COI ≥ 1 and AGAP > 4.038. Negative results: M2BPGi COI < 1 and AGAP ≤ 4.038.

**Table 7:** Validity test for the combined examination of the M2BPGi levels and the AGAP score on the Fibroscan® examination in subjects who have not received or have received antiviral therapy.

	`	Grade Fibroscan (METAVIR Fibroscan)		
		Advanced fibrosis	No/low-grade fibrosis	
Have not received antiviral therapy	M2BPGi + AGAP score	*n* = 9	*n* = 19	Sensitivity: 77.8% Specificity: 100% Positive predictive value (PPV): 100% Negative predictive value (NPV): 90.5% Area under the ROC curve (AUC): 0.889
	Positive	7	0	
	Negative	2	19	
Have received antiviral therapy	M2BPGi + AGAP Score	*n* = 12	*n* = 26	Sensitivity: 83.3% Specificity: 100% Positive predictive value (PPV): 100% Negative predictive value (NPV): 92.9% Area under the ROC curve (AUC): 0.917
	Positive	10	0	
	Negative	2	26	

## Data Availability

All data and tables used to support the findings of this study are included within the article and available upon request to the corresponding author.
